# Post-Acute Sequelae of COVID-19 (PASC) in Pediatrics: Factors That Impact Symptom Severity and Referral to Treatment

**DOI:** 10.3390/children10111805

**Published:** 2023-11-14

**Authors:** Catherine M. Soprano, Ryan Ngo, Casey A. Konys, Ashley Bazier, Katherine S. Salamon

**Affiliations:** 1 Nemours Children’s Health, Wilmington, DE 19803, USA; catherine.soprano@nemours.org; 2 Sydney Kimmel College of Medicine, Thomas Jefferson University, Philadelphia, PA 19107, USA; 3 Bowdoin College, Brunswick, ME 04011, USA; 4 Lurie Children’s Hospital of Chicago, Chicago, IL 60614, USA; 5 School of Medicine, Indiana University, Indianapolis, IN 46805, USA

**Keywords:** PASC, COVID-19, pediatric

## Abstract

The post-acute sequelae of COVID-19 (PASC) is a complex condition. While there are emerging studies on its effects in adults, there is scarce research regarding the long-term effects of COVID-19 infection among youth. Several researchers have likened long-haul COVID-19 to chronic fatigue syndrome/myalgic encephalomyelitis (CFS/ME) and postural orthostatic tachycardia syndrome (POTS). In adults, the prognosis for these diagnoses is less promising than that in youth; however, there is currently very little information available on the presentation of youth with PASC. A better understanding of the specific symptom presentation for youth diagnosed with PASC is necessary. Retrospective chart reviews were conducted collecting demographic data, COVID-19 symptoms and disease progression, and vaccination status. Additional data on referrals to a PASC treatment program and appointments attended were collected. Overall, data suggested that youth present with less severe PASC symptoms than adults, and the role of vaccination is unclear. These youth are often not referred to treatment programs. More exploration is necessary to continue to build an understanding of how best to aid youth diagnosed with PASC.

## 1. Introduction

Since the onset of the novel COVID-19 pandemic, 7.3 to 23 million Americans have suffered from post-acute sequelae of COVID-19 (PASC), reporting symptoms for months to years after testing for COVID-19 is negative [1]. While ongoing research on the phenomenon has been emerging among adult populations, little is known in pediatric populations [2]. Recent studies among adults have found that diagnosing PASC has become increasingly difficult as it is linked to more than 200 symptoms, ranging from malaise, fatigue, and muscular fatigue to autonomic dysfunction [3]. Many of the most common symptoms appear to mimic chronic fatigue syndrome/myalgic encephalomyelitis (CFS/ME) and dysautonomia. Adult studies have linked long-term clinical manifestations of PASC with decreased daily function and overall quality of life [4]. Emerging research is beginning to explore risk factors associated with the development of PASC and the treatment of PASC in adults. Some of these include female sex, the severity of acute COVID-19 infection including the need for invasive or noninvasive mechanical ventilation, the severity of initial respiratory disease as assessed with CAT scans, and the length of hospitalization for COVID-19 infection [5,6]. Risk factors such as the patient’s diet, alcohol use, smoking behaviors, amount of exercise prior to acute COVID-19 infection, and prior sleep patterns may also contribute to the development of PASC [7]. However, PASC research among youth is still in the early stages.

PASC presentation in adults is quite variable. All systems can be involved, but greater than 55% of patients have three or more different symptoms. The most common symptoms in adults are fatigue (53.1%), dyspnea (43.4%), joint pain (27.3%), and chest pain (21.7%) [5]. Pulmonary symptoms often include dyspnea, decreased exercise capacity, and hypoxia. Hematologic sequelae such as thromboembolic events are quite rare (less than 5%), but the duration of the hyperinflammatory state after acute COVID-19 infection is unknown. The most common persistent cardiovascular symptoms include palpitations, dyspnea, and chest pain. There are a number of common persistent neurologic and psychiatric symptoms, including fatigue, myalgia, headache, dysautonomia, and cognitive impairment (brain fog). Anxiety, depression, and post-traumatic stress disorder have been reported to have a rate of 30% to 40% in COVID-19 survivors. Patients who exhibited acute kidney injury during their initial COVID-19 infection may have persistence of reduced eGFR for up to six months. Patients with pre-existing diabetes mellitus may have worsening control of their blood glucose values after an acute COVID-19 infection. Thyroid disease can also be exacerbated in the months following acute COVID-19 infection [5,6]. There are newer reports of a drastic increase in post-COVID-19 development of irritable bowel syndrome (3.2% vs. 0.5%) [8]. Hair loss is the most common dermatologic complaint in PASC. Ormiston and colleagues [9] also linked postural orthostatic tachycardia syndrome (POTS) to PASC, and this has been attributed to the induction of microvascular endothelial dysfunction in patients with acute COVID-19. At this time, it is unknown whether the microvascular endothelial dysfunction contributes in any way to POTS pathophysiology [9].

PASC in youth continues to be less understood. Ongoing research into the risk and protective factors of PASC for children and adolescents is needed. In a recent study, about 17% of the sample of children and adolescents who had a COVID-19 infection met the criteria for PASC, with older age and obesity noted as risk factors [10]. Additionally, the same study noted that respiratory symptoms were reported early in the PASC presentation, and neurocognitive symptoms became more common the longer the symptoms persisted. There remains much speculation as to why this pattern has emerged. Disease severity, and subsequent deconditioning as a result of inactivity, appear to be the main hypothesis. However, vaccination status and other social determinants of health (e.g., socioeconomic status, access to healthcare, transportation, etc.) may also play a role.

A variety of socioenvironmental factors have been found to predispose adults to PASC [11]. Vaccination status for COVID-19 at the time of the initial COVID-19 infection is one of the factors explored. A 2022 CDC Household Pulse Survey [12] on 62,000 Americans concluded that adults in states with the lowest vaccination rates (Wyoming, Mississippi, Louisiana, and Idaho) yielded the highest numbers of self-reported PASC cases, between 16.1% and 21%. On the other hand, states with the highest vaccination rates (Rhode Island, Maine, Vermont, and Massachusetts) had comparably lower self-reported PASC cases, between 6.7% and 12.8%. Exploring if vaccination status is also a contributing factor to the development of PASC among youth will be vital to aid with ongoing education and recommendations. Cumulative data from the CDC outline racial disparities within COVID-19 hospitalizations and infections. Age-standardized data demonstrate that, on average, Black, Hispanic, Indigenous/Alaskan Native, as well as Native Hawaiian and Pacific Islanders, have about a one-and-a-half times higher risk of contracting COVID-19 than white people and are also twice as likely to die from COVID-19 compared with their white counterparts [11].

It is important to note that the COVID-19 pandemic has highlighted and exacerbated existing healthcare disparities. Research has found disparities in COVID-19-positive cases, vaccination rate, and access to vaccinations related to several social determinants of health. For example, research has found that racially minoritized youth experienced higher rates of infection and hospitalization [13]. Disparities also exist in vaccination rates with racially minoritized youth, younger children, and children from rural areas having lower vaccination rates [14]. There are several possible explanations for these disparities, including poverty rates, lack of access to consistent healthcare, and vaccine hesitancy due to historical events in medical history. Given these disparities and the findings that vaccination status may be a contributing factor to PASC development, it is important to consider the potential impact of sociodemographic factors and healthcare inequities in PASC development in youth.

Herrera and colleagues [15] reported on the American Academy of Physical Medicine and Rehabilitation (AAPM&R) Multi-Disciplinary PASC Collaborative that was created to address the growing concern about how to address patients with PASC. Recommendations highlight the need to address the physical manifestations of PASC by using a slow introduction of physical activity, healthy habits (e.g., hydration, sleep, etc.), and energy conservation strategies. The AAPM&R emphasizes the similarities between PASC and CFS/ME and dysautonomia. Many adult hospitals are utilizing the current evidence-based treatment for both CFS/ME and dysautonomia to guide treatment recommendations for PASC [16]. This may include medical evaluations and testing through physician specialists based on symptom presentation as well as involvement with physical therapy, nutrition, and psychological services to address the ongoing nature and impact of the symptoms.

Because of the lack of research on youth with PASC, this study aimed to better understand the spectrum of symptoms reported by youth with PASC and attempt to compare these symptoms with current reports on adult populations in the literature with the hypothesis that youth with PASC would present with similar symptom presentations as adults. Additionally, data were utilized from the electronic medical record for exploratory analyses to examine any associations with socioenvironmental factors, vaccination status, and PASC diagnosis as well as whether any of the specific symptoms were positively or negatively affected by vaccination status. Lastly, the hospital system in which the data were collected had an interdisciplinary pain program that served as the treatment referral source for youth with PASC. The study aimed to gather preliminary data on treatment design and functional impairment.

## 2. Materials and Methods

### 2.1. Procedure

An IRB-approved (approval date: 14 February 2022) retrospective chart review was conducted on patients aged 7 to 21 years who presented to the health system (all locations) and for whom a diagnosis of “post-COVID-19 condition, unspecified” (ICD-10 code: U09.9) within the electronic medical record (EMR) was indicated. Inclusion criteria include any youth between 7 and 21 years old who presented to the health system and was diagnosed with PASC. Youth were excluded if they did not meet the age requirements. No other exclusion criteria were utilized.

Once patients were identified through a data pull from the EMR, an undergraduate student and medical student reviewed the patients’ charts to collect information on COVID-19 symptoms, infection dates, vaccination dates (if applicable), and other demographic data. Information on patient outcomes (see Section 2.3) was linked via medical record number and patient name. Data were collected manually using Microsoft Excel and then exported to a data management system for further analyses (see Section 2.4).

### 2.2. Integrated Pain and Wellness Program

The Integrated Pain and Wellness Program is an outpatient, interdisciplinary clinic for youth experiencing chronic pain and other physical symptoms. Youth patients receive individualized treatment plans. These include weekly cognitive behavioral therapy (CBT) sessions with a licensed pediatric pain psychologist or a supervised trainee in psychology, weekly or twice weekly physical and/or occupational therapy, and/or integrative therapies (i.e., healing touch, yoga, and massage). A physician or advanced practice nurse provides medication management as necessary. Youth can be referred for any symptom or pain leading to functional impairment.

A subset of youth patients were referred to the Integrated Pain and Wellness Program within the hospital system. This program offers interdisciplinary treatment for chronic pain and physical symptoms, such as those experienced in PASC. For those who were referred to the Integrated Pain and Wellness Program, program data included the total number of appointments with each of the disciplines. These disciplines included physical therapy, occupational therapy, psychology, and integrative medicine, which includes massage, healing touch, yoga, and nutrition (for the purposes of this study).

Youth and their parent/guardian who attended the initial appointment with the Integrated Pain and Wellness Program were invited to complete a series of questionnaires electronically via REDCap [17,18] and an invitation to complete the questionnaires at three months following the initial evaluation was sent electronically via email. For the purpose of this study, the questionnaires on pain interference and anxiety were utilized.

### 2.3. Measures

*Demographic and health information*, including age, race, ethnicity, biological sex and gender identity, and zip code, were collected. COVID-19 infection dates, vaccination dates, and symptom presentation were collected from the EMR.

The *PROMIS Pediatric Pain Interference Scale* [19] is an eight-item self-report questionnaire assessing the extent to which pain interferes with functioning (e.g., emotional, academic, physical) over the past 7 days. Youth rate items based on a Likert scale from 1 (never) to 5 (almost always). Raw scores are transformed into T-scores. Higher scores indicate greater levels of pain interference. This scale has strong psychometric properties [19].

The *PROMIS Youth Anxiety Short Form* (PROMIS-YA SF) [20] is an eight-item form derived from the item bank for Pediatric Anxiety developed by the NIMH-sponsored PROMIS. Items assess fear worry and hyperarousal over the past week. On a scale from 0 (never) to 4 (always), youth rate how often they experience these feelings. Scores are transformed into T-scores. Higher scores indicate greater levels of anxiety.

### 2.4. Data Analysis

Analyses were conducted in SPSS, version 27 (IBM Corp., Armonk, NY, USA). Descriptive statistics were utilized to examine the demographic data collected. Pearson’s chi-square test was used to evaluate the differences between demographic characteristics and social determinants of health (e.g., vaccination status), symptom presentation, and referral status to the Integrated Pain and Wellness Program. Additionally, *t*-tests were planned to compare PROMIS Pain Interference and PROMIS Anxiety at the initial evaluation and three-month post-evaluations for youth who were referred to the Integrated Pain and Wellness Program.

## 3. Results

### 3.1. Patient Characteristics

Between March 2020 and October 2022, 303 youth patients received a PASC diagnosis across all hospital locations in the EHR healthcare system. On average, youth were white (67%), non-Hispanic (79%), and 13 years old with a female identity (55%). Table 1 lists additional demographic characteristics.

**Figure 1:**
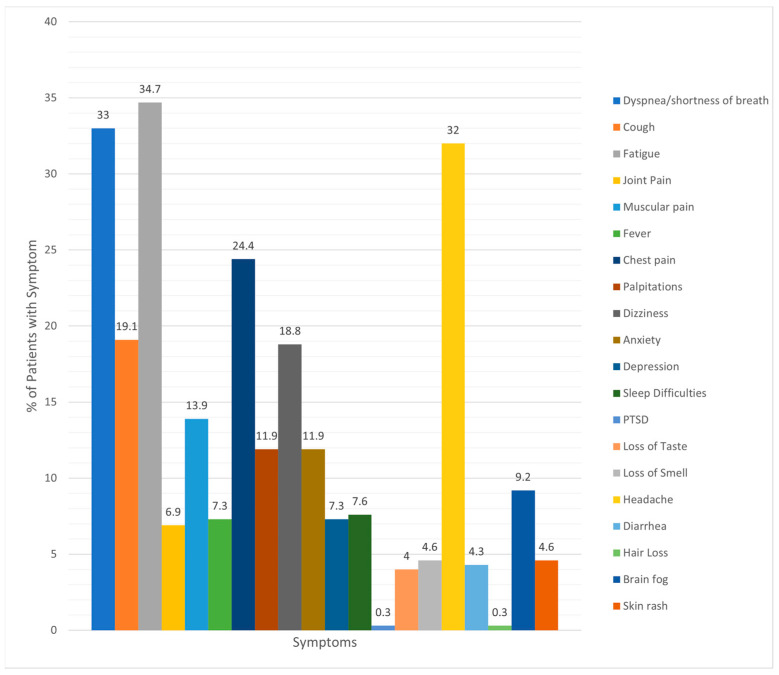
Incidence of various symptoms reported by patients with PASC.

About half of the youth lived in Florida (155, 51.2%) and the surrounding area, while the remainder lived in the Delaware, New Jersey, and Pennsylvania tri-state region (referred to as the Delaware Valley; 148; 48.8%). Figure 1 illustrates PASC symptom presentation at the time of the diagnosis. Shortness of breath, fatigue, headache, and chest pain were the most common symptoms.

### 3.2. Vaccination Status

Of the 75% of youth eligible to receive the COVID-19 vaccination based on CDC guidelines, only 31% of youth received the initial COVID-19 vaccination, with 8% receiving a subsequent recommended booster. There were no differences in vaccination rates between youth living in Florida (45 of 116 eligible) and Delaware (50 of 111 eligible). Youth who were vaccinated tended to report less shortness of breath as part of the PASC symptom presentation, although this was not statistically significant. No other differences in vaccination status and symptoms were noted (Table 2).

Vaccination status was not different between biological sex, gender, race, or ethnicity (Table 3). There was a significant difference when compared with age (older youth tended to be vaccinated).

### 3.3. Integrated Pain and Wellness Program Referrals

As the program is only located in the Delaware Valley, youth with PASC living in Florida were excluded from these analyses. Of the 148 patients in Delaware Valley diagnosed with PASC, 27 (18.4%) were referred to the Integrated Pain and Wellness Program for treatment. Of those 27 youth patients, 23 attended an initial evaluation within the program, and all started treatment. Youth who reported fatigue, joint pain, muscle pain, dizziness, anxiety, depression, and brain fog as part of the PASC presentation were more likely to be referred to the program. As seen in Table 4, youth attended a wide range of appointments within the treatment program. These appointments included physical and occupational therapy, psychological services, and integrative medicine with plans of care ranging from zero follow-up appointments to up to 30 total physical therapy sessions.

Of the six caregivers and three youth who completed the measures on symptom interference, all noted clinically significant levels of impairment at the initial evaluation. Caregivers tended to report higher levels of anxious symptoms for teens. Because of the small sample size, no additional statistics were completed. Additionally, no three-month follow-up questionnaires were received; thus, there were no data available for comparison.

## 4. Discussion

Overall, this study revealed that most of the youth in this sample were not vaccinated against COVID-19, and vaccination status did not differ based on location, gender, biological sex, race, or ethnicity. Based on the data gathered in this study, it is difficult to determine if vaccination status attenuated symptom presentation in PASC. It was observed that youth who were vaccinated tended to experience less shortness of breath as part of the PASC presentation. This may be related to COVID-19 infection severity. However, due to the retrospective nature of this study, no information on the acute COVID-19 infection was available. More investigation is necessary to explore how vaccination status and disease severity at the time of the infection may attenuate or lessen the risk of the development of PASC.

During this study period, approval for the COVID-19 vaccination for youth was actively occurring. The dates for approval were as follows: Those 16 years of age and older were approved on 12 November 2020, those 12–15 years old were approved on 20 May 2021, and those 5–11 years old were approved on 29 October 2021. Thus, the low vaccination rate could have been confounded by the lack of approval and access to the vaccine for certain age groups. Ullah and colleagues (2021) also reflected on multiple myths and misperceptions about the COVID-19 vaccination that may have influenced vaccination rates. There are other examples of vaccination hesitancy in the United States, specifically focused on measles and recent outbreaks in California. Fear of side effects and anti-vaccination endorsements on social media have been noted to influence others [21]. Additional research is needed to explore families’ attitudes toward vaccination at this time. Knowing this information, as well as the role of vaccinations in attenuating long-term effects of illness severity, like PASC, could be beneficial to promote vaccinations in youth and families experiencing hesitancy. It is unclear if vaccination status may be protective, but more studies are necessary.

Anecdotally, when comparing the symptom presentation of youth with PASC with published adult data, symptom presentation appears similar, although less severe in youth. Joint pain may be relatively less common in youth than in adults [3]. As compared to the review by Wang and colleagues [3], interestingly, headache was much more common in youth diagnosed with PASC (32%). One explanation could be that the acute COVID-19 infection is often less severe in youth overall. Youth may not have been bed-bound as long or restricted in activities for an extended period, potentially allowing for a speedier recovery from the acute infection and less severe PASC symptoms overall. While there are many symptom similarities of PASC between adult and youth populations, it is important to continue to explore the exceptions. These similarities could aid with the awareness of this condition in pediatrics to expedite referrals and access to treatment.

According to the World Health Organization (WHO), the prevalence rate of PASC (defined as experiencing ongoing symptoms at 90 days post-infection) is 2% to 10% for youth but could be as high as 25% depending on criteria utilized to define PASC [22]. While the current study did not compare all youth diagnosed with COVID-19 with those diagnosed with PASC within the healthcare system, approximately 18% of youth were referred to a treatment program for PASC, which could be an indication of PASC severity given that these youth and families were struggling with symptoms. The WHO recently developed a consensus definition of pediatric PASC to aid with identification, diagnosis, and treatment. This definition includes the presence of one or more new physical symptoms (relapsing or chronic) and the length of at least 12 weeks after confirmed initial SARS-CoV-2 infection. The symptoms must also lead to functional impairment. This consensus in symptoms will aid with the overall recognition of PASC in youth and could lead to more referrals to treatment programs that focus on functional improvements and quality of life.

As such, only a small portion of youth diagnosed with PASC were referred to a treatment program within the hospital system despite the program advertising itself as equipped to evaluate and treat youth with PASC. Rao and colleagues [2] highlighted that the incidence of pediatric PASC in a large-scale study was low, thus suggesting that these data are more representative of incident rates. Alternatively, it is well known in the literature that adults and youth with chronic pain often experience stigma when seeking care. While Freund and colleagues [23] noted that adults with respiratory symptoms following COVID-19 did not have major symptom changes between 3- and 6-month follow-ups, youth with post-acute COVID-19 symptoms that affect day-to-day activities may benefit from a rehabilitation treatment approach. More recently, Gorna and colleagues [24] highlighted the same type of stigma for adults with symptoms of long-haul COVID-19. In a recent study in Finland, researchers noted that PASC was rarely diagnosed in the primary care setting, with suggestions that lack of knowledge about PASC is the main barrier [25]. Taken together, improved education about the potential development of PASC in youth and common symptom presentation and prevalence rates could lead to more accurate diagnosis for youth struggling with these symptoms. Educational programs could target primary care and pediatrician offices as youth likely present to these locations with the first signs and symptoms of PASC following a COVID-19 infection. These clinicians could be at the front line of providing accurate diagnoses of PASC and helping families find treatment programs. Early screening to identify PASC symptoms could be universally administered to any youth presenting to a primary care visit following a COVID-19 infection and may be more necessary in youth than in adult populations [26].

A biopsychosocial treatment approach [27] is necessary for the treatment of PASC in order to address the biological, psychological, and sociocultural impact of COVID-19. Additionally, advocacy for PASC programs has been suggested in order to include the voices of patients and families who have lived experiences with COVID-19 and PASC. Greater awareness that children and adolescents can develop PASC is necessary. It is clear that youth experience a constellation of symptoms that vary widely, with a significant impact on their day-to-day life. Additionally, mental health and neurocognitive symptoms are often reported, which may lead to more significant long-term disability if not addressed early.

This study has several notable limitations. First, only youth within the system with a PASC diagnosis were evaluated. This may have impacted the heterogeneity of the sample, including the limited age range and other demographic characteristics. Future research should compare all known COVID-19 infections within the hospital system to understand the potential risks and protective factors for the development of pediatric PASC. Because of the low sample size referred to the treatment program, exploratory statistics to determine the effectiveness of the program were not possible. All the youth and their families who presented to the program experienced significant functional impairment; thus, more research on effective treatments to improve quality of life is necessary. Unfortunately, access to and completion of optimal treatment were not able to be assessed in this study. Lastly, as this study was a retrospective chart review, it was not possible to determine if more youth with a history of SARS-CoV-2 infection were underdiagnosed or dismissed and did not receive a PASC diagnosis within the EHR. While the prevalence of PASC in past research aligns with these data, it could be that PASC itself is underdiagnosed in youth, leading to delays in treatment.

Overall, this study highlights the susceptibility of youth who have had COVID-19 infection to PASC. Symptom presentations are similar to those in adult populations with PASC but do have some notable differences. It is unclear from the current data if vaccination status attenuated PASC symptoms. Most youth diagnosed with PASC were not referred to additional treatment. It is unknown how many other youth within this hospital system would have met the criteria for a PASC diagnosis but did not receive one. This suggests that more awareness of PASC in pediatric populations is necessary.

## 5. Conclusions

Symptoms of post-acute sequelae of COVID-19 (PASC) in youth are similar to adults with a notable increased incidence of headaches and decreased incidence of joint pain in youth populations. Little continues to be known about PASC in youth, and PASC may be underdiagnosed in youth populations. More studies to understand PASC in youth globally and specific reasons for why underdiagnosis occurs may aid in further understanding of this disease phenomenon and treatment approaches. Because of small numbers, no conclusions can be made about treatment effects.

## Figures and Tables

**Table 1:** Demographic characteristics.

Gender Identity	%
Female	55.4
Male	44.2
Nonbinary	0.3
Race	%
White	67
Black	14.9
Asian	2.6
Other Pacific Islander	0.3
Mixed/Biracial	0.3
Other PI	14.9
Ethnicity	%
Hispanic	20.8
Non-Hispanic	79.2

**Table 2:** Vaccination status and presentation of symptoms.

Symptom	Vaccinated/ Symptom Present	Vaccinated/ Symptom Not Present	Not Vaccinated/ Symptom Present	Not Vaccinated/ Symptom Not Present	*p*-Value
Dyspnea/ shortness of breath	37	58	51	135	0.49
Cough	21	74	30	156	0.219
Fatigue	35	60	66	120	0.822
Joint pain	8	87	12	174	0.544
Muscular pain	12	83	30	156	0.437
Fever	5	90	16	1370	0.314
Chest pain	21	74	43	143	0.848
Palpitations	8	87	24	162	0.263
Dizziness	19	76	32	154	0.565
Anxiety	16	79	19	167	0.112
Depression	10	85	12	174	0.229
Sleep difficulties	7	88	16	170	0.721
PTSD	0	95	1	185	0.474
Loss of taste	3	92	8	178	0.64
Loss of smell	3	92	11	175	0.315
Headache	31	64	58	128	0.805
Diarrhea	3	92	10	176	0.402
Hair loss	0	95	1	185	0.474
Brain fog	7	88	18	168	0.52
Skin rash	6	89	8	178	0.463

**Table 3:** Differences in vaccination status.

		Fully Vaccinated	Not Fully Vaccinated	
Age (mean)		14.44 (2.99)	12.93 (3.24)	F(2) = 7.60, *p* = 0.001
Biological sex at birth				χ^2^(2) = 5.68, *p* = 0.058
	Male	40	78	
	Female	55	108	
Gender				χ^2^(4) = 6.11, *p* = 0.191
	Male	40	79	
	Female	55	106	
	Nonbinary	0	1	
Race				χ^2^(10) = 13.52, *p* = 1.96
	White/Caucasian	64	122	
	Black/African American	13	31	
	Asian	6	2	
	Other Pacific Islander	0	1	
	Mixed Race/Biracial	1	0	
	Other	11	30	
Ethnicity				χ^2^(2) = 1.99, *p* = 0.370
	Hispanic	21	40	
	Not Hispanic	74	146	

**Table 4:** Treatment plan and initial PROMIS scores.

Treatment Type	# of Visits	SD	Range
Physical therapy	3.87	6.46	0–30
Occupational therapy	0.61	1.58	0–7
Integrative medicine	1.17	2.01	0–8
Psychology	2.75	2.61	0–10
PROMIS Anxiety (T-score)	Caregiver	Teen	
	45.4	43.5	
	54	45.1	
	55.8	54	
	70.5		
	85.2		
	85.2		
PROMIS Pain Interference (T-score)	Caregiver	Teen	
	63	59.9	
	64.2	61.5	
	66	68.9	
	74.2		
	77.6		
	77.6		

## Data Availability

The data presented in this study are available on request from the corresponding author. The data are not publicly available due to privacy and HIPPA.
